# Supplementary material for: Rapid construction of a whole-genome transposon insertion collection for Shewanella oneidensis by Knockout Sudoku
Source: Nat Commun. 2016 Nov 10;7:13270. doi: 10.1038/ncomms13270 (PMC5109470; doi:10.1038/ncomms13270)
Supplement: Supplementary Information — Supplementary Figures 1-8, Supplementary Tables 1, Supplementary Notes 1-3, Supplementary References. [file ncomms13270-s1.pdf]

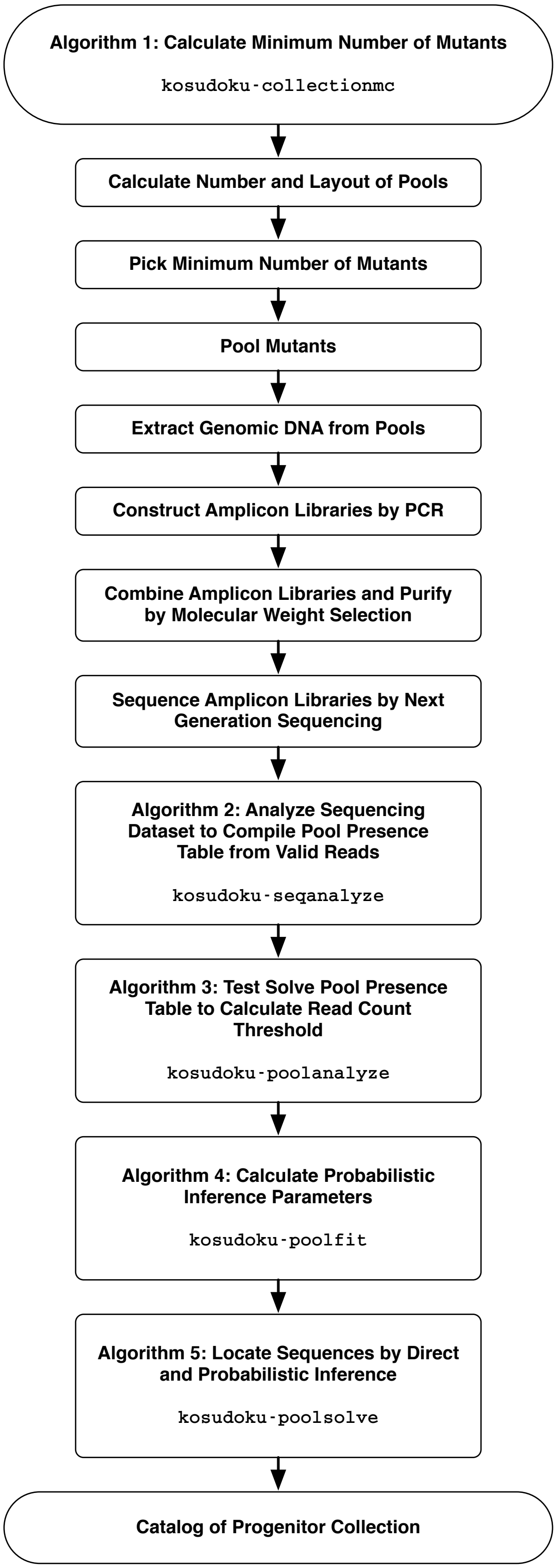

Supplementary Figure 1: Flow chart for creation and annotation of progenitor collection (**Methods**).

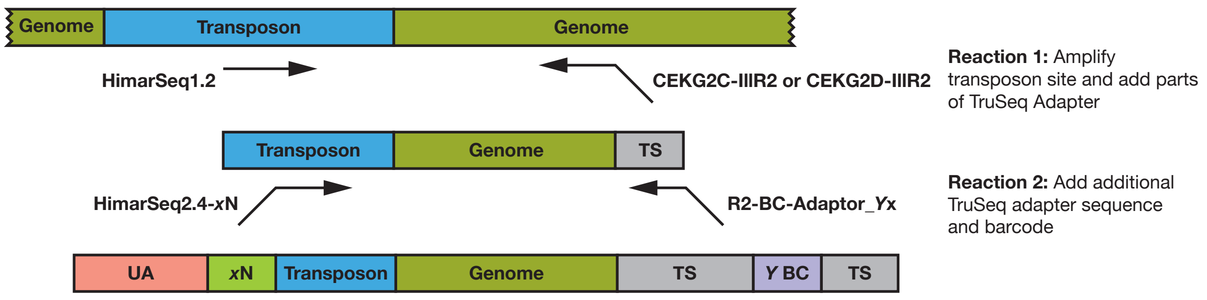

Supplementary Figure 2: Schematic of nested PCR reaction for amplicon library generation from the genomic DNA of pooled transposon insertion mutants. UA: Illumina Universal Adaptor sequencing-primer- and flow-cell-binding sequence; *x*N: a random 4-7 bp sequence needed for Illumina camera focusing; TS: Illumina TruSeq flow-cell- and sequencing-primer-binding sequence; BC: custom barcode sequence unique to each pool (*Y* = 0-60) in place of the standard Illumina index sequence. Primer sequences are listed in **Supplementary File 5** and protocol is listed in **Methods**.

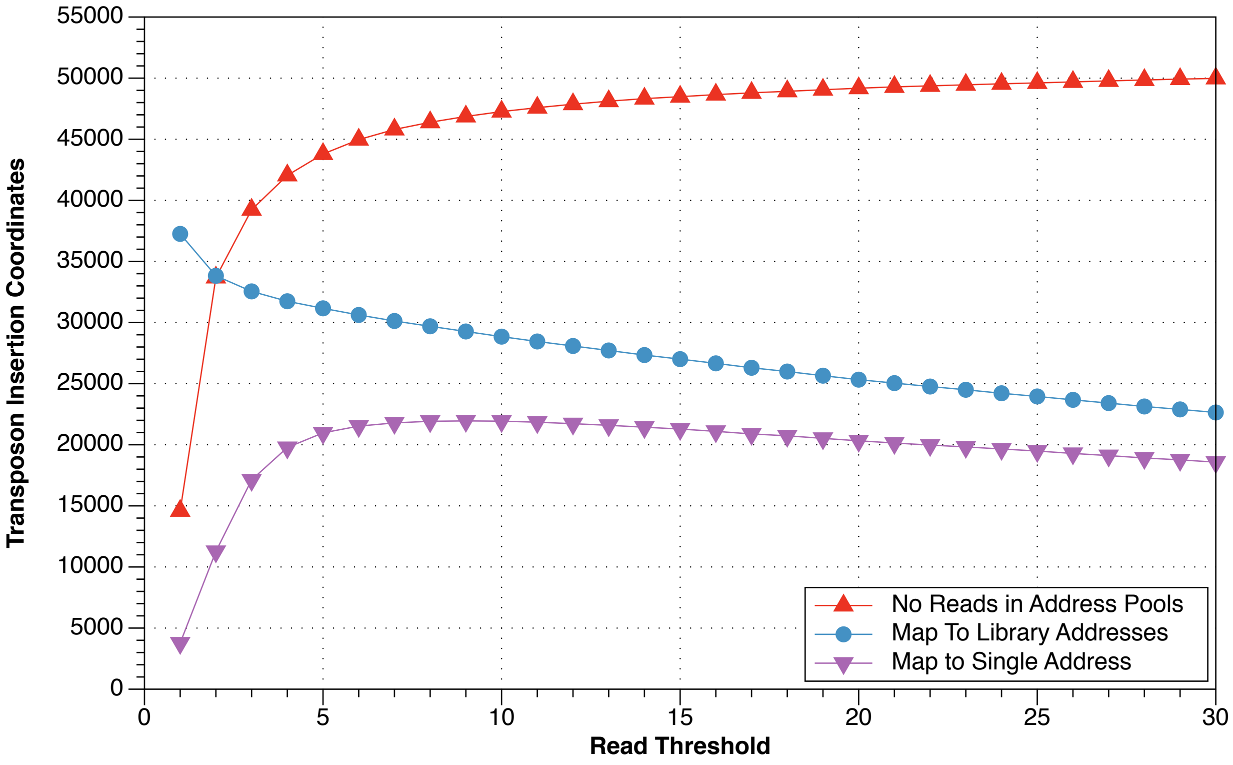

Supplementary Figure 3: Stability of Knockout Sudoku progenitor collection solution. The plot shows how the solution to the progenitor collection varies as a function of the read count threshold needed for a coordinate to be used in mapping a transposon to a location in the collection. Red triangles show how the number of pool presence table lines (transposon coordinates) with no address coordinates begins to plateau after the read count threshold reaches 5. Purple inverted triangles show how the number of transposon coordinates that map to single addresses plateaus at a read count threshold of 5.

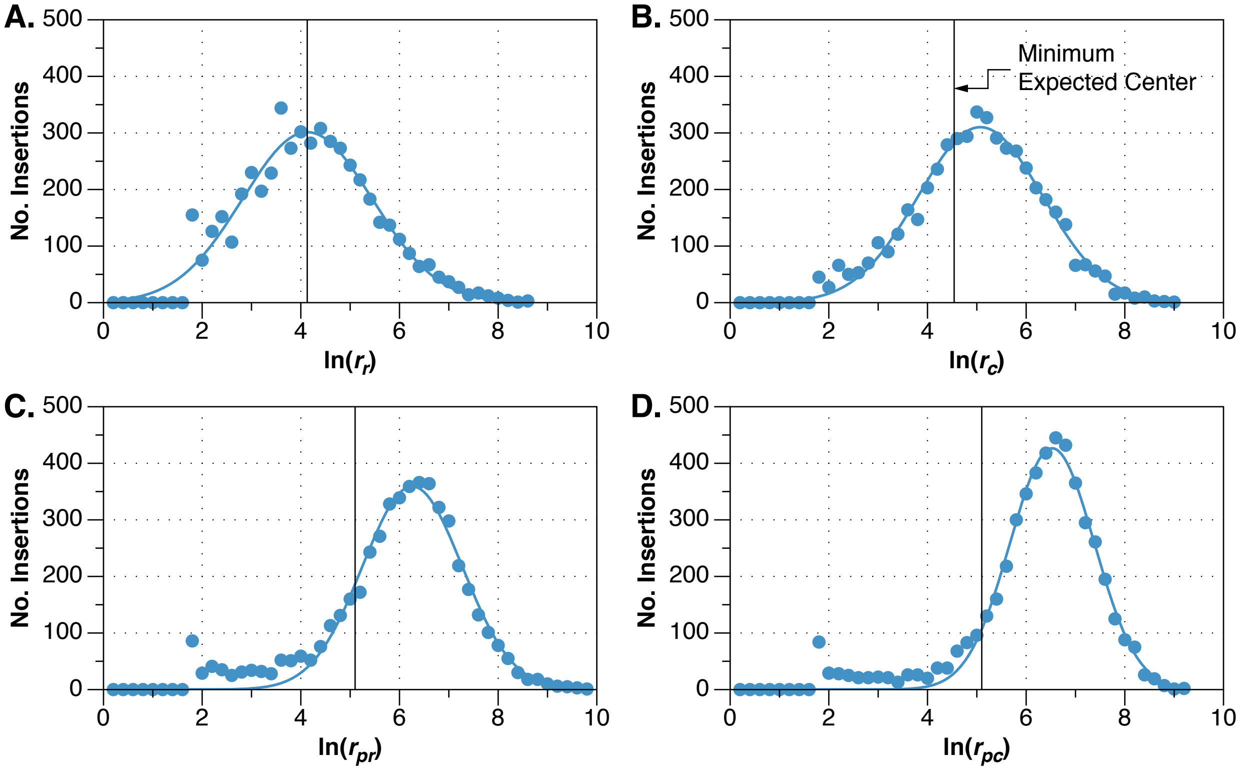

Supplementary Figure 4: Distribution of read counts associated with coordinates in lines from the pool presence table that map to single addresses (e.g. **Fig 3B, lines 1 and 2**). **A:** all row pools; **B:** all column pools; **C:** all plate row pools; **D:** all plate column pools. Closed blue circles are number of lines, blue lines are Gaussian best fits. The minimum expected position of the center of the column, plate row and plate column read count distributions relative to the center of the row read count distribution are indicated by vertical lines on panels **B**, **C** and **D** (**Equations S19, S20** and **S21**).

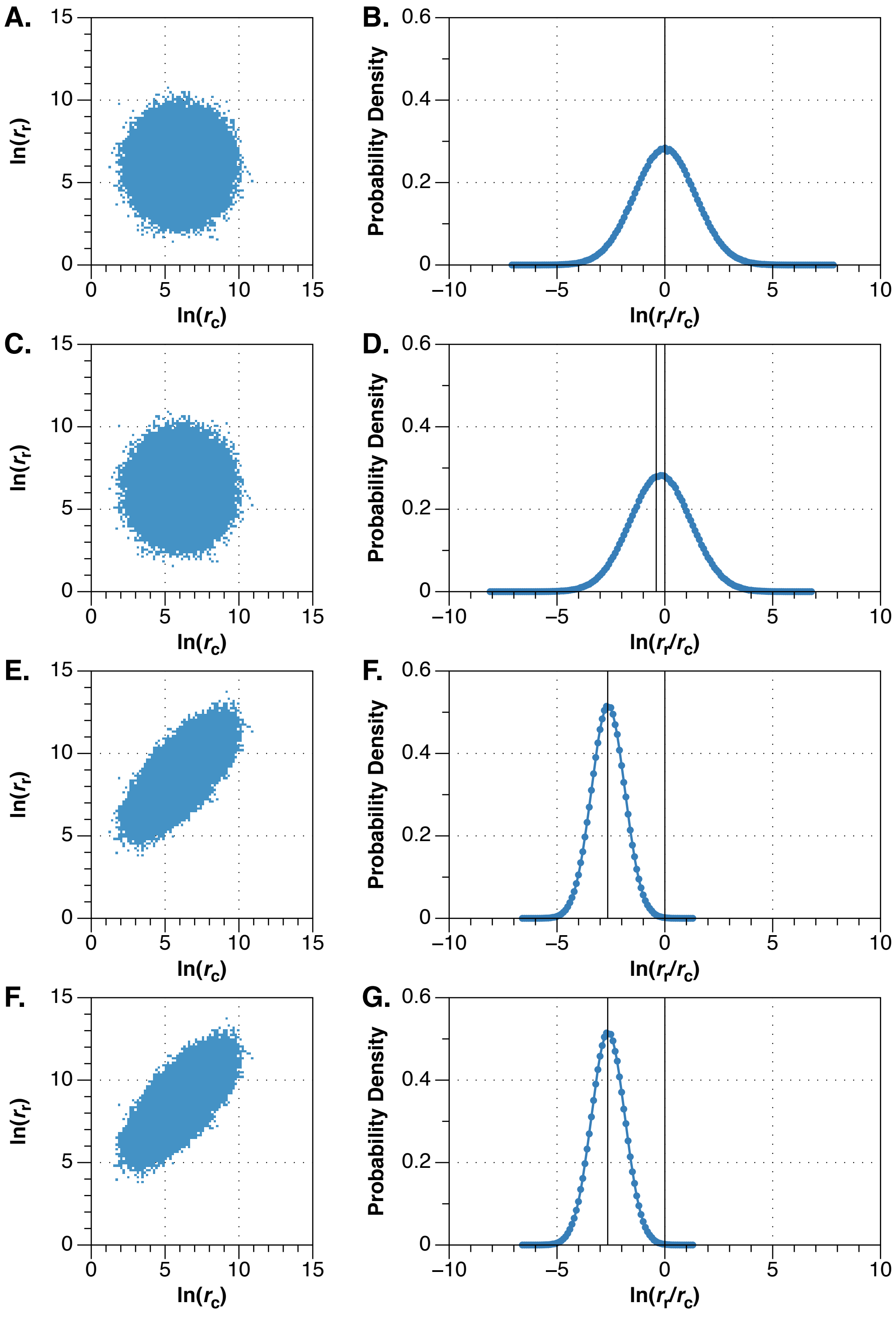

Supplementary Figure 5: Ratios of two random distributions as correlation is increased, and finally thresholding is added. **A** and **B**: two uncorrelated random distributions (*r* and *c*) that share the same mean and standard deviation. **C** and **D**: the mean of *c* is increased to 1.5× that of *r*. **E** and **F**: Correlation of *r* and *c* is further increased by application of a covariance matrix with non-zero off-diagonal elements. **F** and **G**: a threshold is applied to *r* and *c* that removes any pair of numbers that are not above 5.

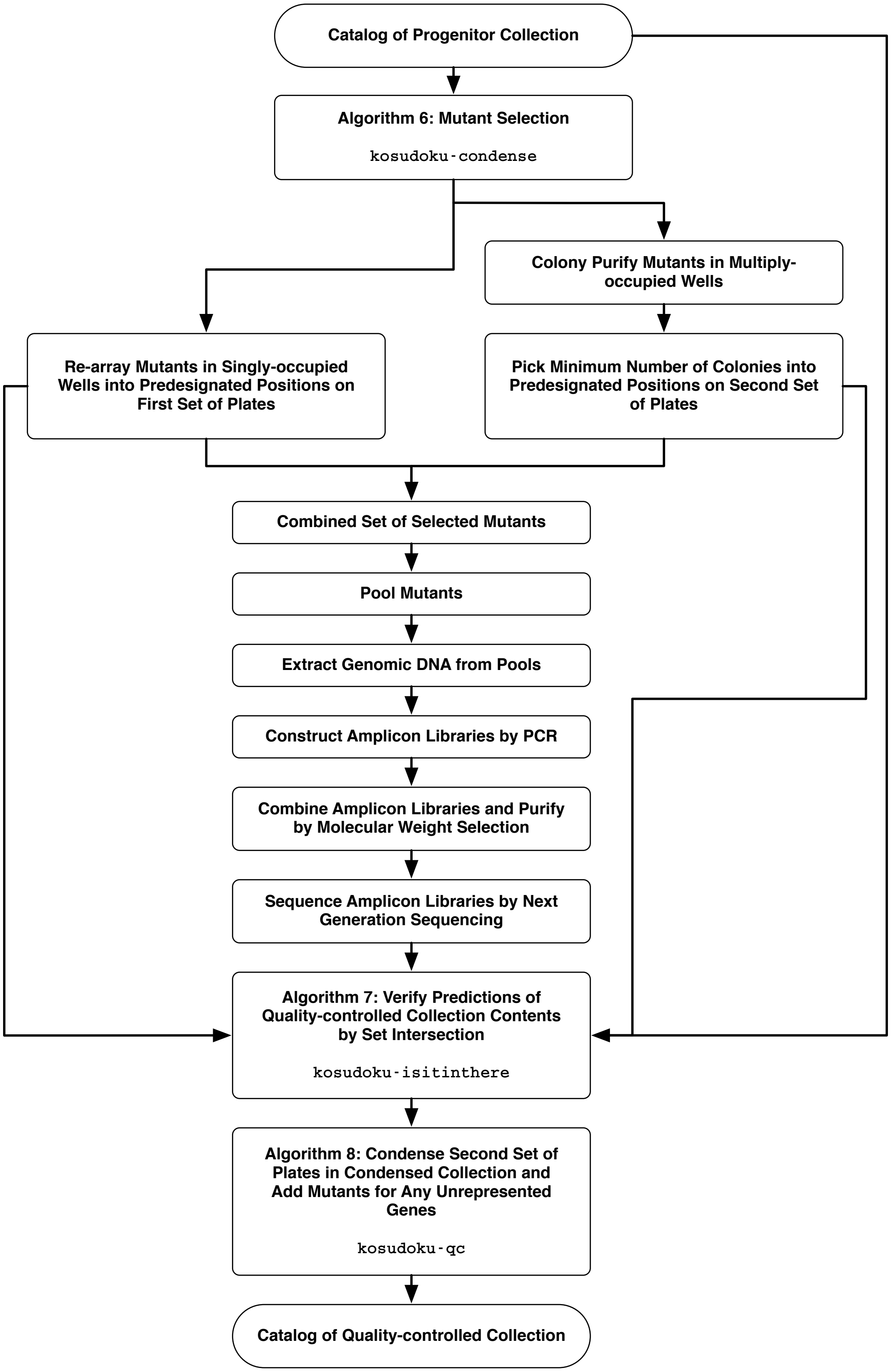

Supplementary Figure 6: Flow chart for creation and validation of quality-controlled collection. See **Methods**.

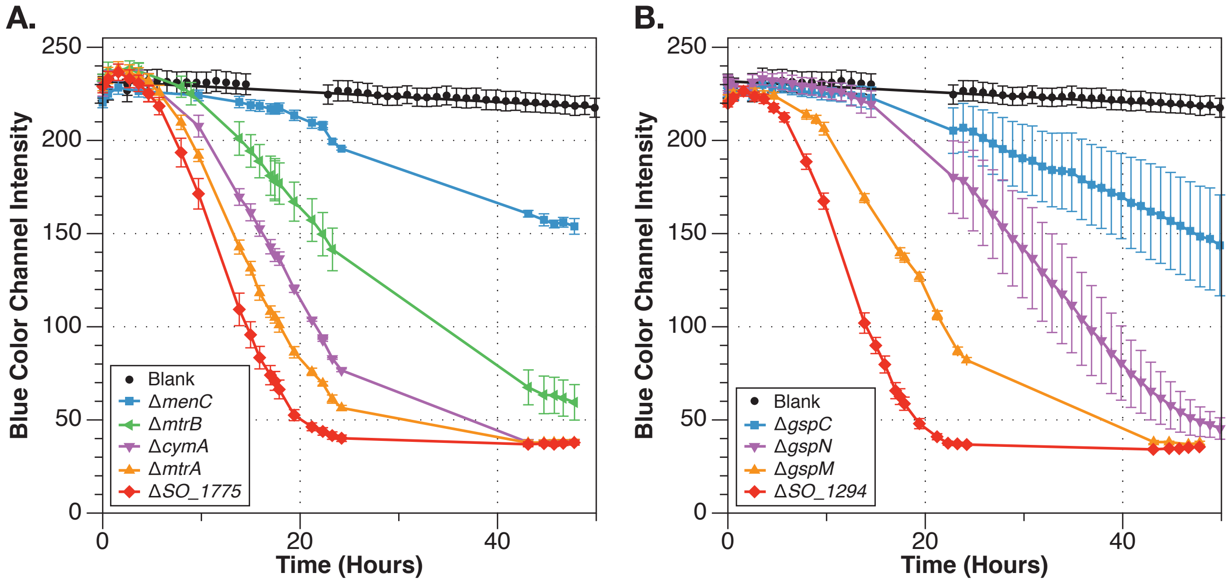

Supplementary Figure 7: A: and **B:** Time course of the reduction state of AQDS indicated by blue color channel intensity for selected mutants. A lower blue intensity indicates greater reduction of the dye (**Methods**).

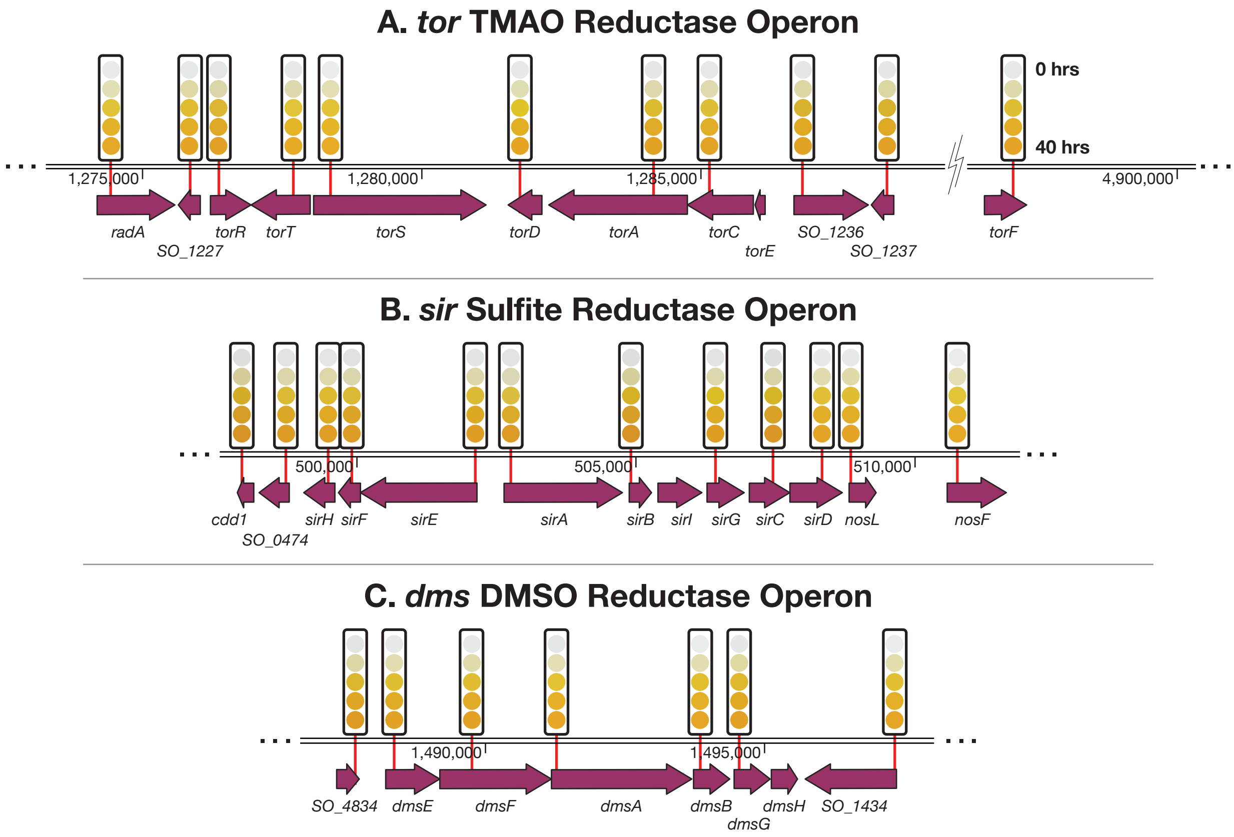

Supplementary Figure 8: AQDS reduction screening results for the TMAO (**A**), sulfite (**B**) and DMSO (**C**) reductases. The state of the AQDS dye at 10 hour intervals is indicated by the series of color circles above the gene (**Methods**). The location of the transposon chosen to disrupt the gene is marked as a red line.

Item	Number	Unit Cost	Total Cost	Note
Illumina HiSeq Sequencing	2	$1,800.00	$3,600.00	
Liquidator 96-channel Pipettor Tips	417	$4.38	$1,826.46	For pooling progenitor collection.
Liquidator 96-channel Pipettor Tips	84	$4.38	$367.92	For pooling quality-controlled collection.
Rental of CP7200 Colony Picking Robot	1	$3,000.00	$3,000.00	
96-well Plates	417	$1.75	$729.75	For progenitor collection
96-well Plates	84	$1.75	$147.00	For quality-controlled collection.
Air Porous Membranes	501	$0.77	$385.77	
Aluminum Plate Sealing Film	501	$1.04	$521.04	
Lids for Microplates	501	$1.09	$545.29	
Barcoding Primers	61	$5.00	$305.00	
OneTaq Polymerase	1	$130.00	$130.00	
		Total Cost	$11,558.23	

Supplementary Table 1: Approximate costs of Knockout Sudoku collection production. A largely complete listing of consumables and equipment used in generation of the *S. oneidensis* Sudoku Collection.

Supplementary Note 1: Comparison of Major Costs in Knockout Collection Generation Methods

When we first identified the need for a *Shewanella oneidensis* whole genome knockout or disruption mutant collection, we looked at several methods available at the time and assessed our ability to perform them given our available budget and time.

There is no doubt that the targeted deletion collections (Yeast Knockout Collection, Keio Collection for *E. coli*) set the gold standard for completeness, precision of gene deletion and reliability of the inferences that can be drawn from genetic screens using them. However, the construction costs associated with these methods are extremely high. Giaever and Nislow[1] estimate that the Yeast Knockout Collection cost $2.31 million ($3.39 million in 2016 dollars) and approximately 4 years to construct.

The construction of each mutant in the Keio Collection required the synthesis of 170 base pairs of unique DNA[2]. Assuming a similar procedure was used to construct the *S. oneidensis* Knockout Collection; that we needed to make ≈ 4,000 gene deletion mutants; and a synthesis cost of $0.35 per base pair, we would have needed to spend $238,000 on DNA synthesis alone. While we feel confident that this figure could be reduced, we were not confident that it could be brought within our budget.

We then considered cataloging and condensation of a saturating coverage transposon mutant collection. However, in order to achieve a high degree of coverage of the *S. oneidensis* genome, we estimated that we would need to sequence at least 40,000 mutants on 417 96-well plates. Given that the semi-nested PCR reaction used to generate amplicons to identify each mutant requires approximately 6 hours of PCR cycler time[3], and that we had at most two 96-well PCR cyclers available to us, we would have needed to run these machines continuously for 1,250 hours (52 days); since this reaction requires human supervision, this may have increased to almost 150 days. Additionally, as each sequencing reaction costs approximately $7, the total sequencing cost of this project would amount to approximately $280,000. Additionally, as each semi-nested PCR reaction uses 4 units of OneTaq DNA polymerase, we would have had to use 160,000 units of polymerase at a total cost of $21,000. Again, while we had confidence that we could reduce these numbers, and that people who have employed this technique have, we did not believe that we could achieve the almost 100-fold cost reduction that we would need to bring this method inside of our budget.

Compared with the previous approaches, the combinatorial pooling method designed by Goodman *et al.*[4,5] was highly attractive. The use of a single next-generation sequencing experiment reduces sequencing costs from ≈ $280,000 to at most ≈ $1,800. However, the time needed to prepare a sequencing library through this method proved to be prohibitive. Goodman *et al.*[5] note that pooling a single plate by their method requires ≈ 1.6 hours with an Eppendorf epMotion liquid handler. When considering options for the *S. oneidensis* Sudoku collection, we considered a similar robot (it was the only robot on the Princeton campus that was publicly available), and concluded that a plate would take approximately 2 hours to pool. This suggests that pooling the 417 plate progenitor collection for the *S. oneidensis* Sudoku collection would require between 667 to 834 hours, or 28 to 35 days of continuous robot operation. As this requires supervision, this would likely take 83 to 104 days, assuming no errors. This time is also expensive: at a user fee of $40 per hour, this process would have cost between $26,688 to $33,360 with an additional $4,587 for speciality pipette tips.

By contrast, with a manual pipettor and a team of 5 people, we were able to pool the 417 plate collection in only a single day, with a pipette tip cost of ≈ $1,800 and a user fee of pizza for the team. This represents a 100-fold (real) time saving and ≈ 20-fold cost saving.

Supplementary Note 2: Calculation of Expected Average Mutant Read Count Ratios

This supplementary note explains how we compute the expected average ratio of read counts per mutant species between pools (**Supplementary Fig. 4**).

When pooling the *S. oneidensis* Sudoku Collection, we withdrew 10 µL of culture from each well. Thus, as each plate has 12 wells per row, the total volume of each row pool,

$$V_r = 417 \times 12 \times 10\,\mu\text{L} = 50{,}040\,\mu\text{L}\,, \tag{S1}$$

while the number of samples in each row pool,

$$S_r = 417 \times 12 = 5{,}040\,. \tag{S2}$$

Similarly, for the column (*c*), plate row (*pr*) and plate column (*pc*) pools,

$$V_c = 417 \times 8 \times 10\,\mu\text{L} = 33{,}360\,\mu\text{L}\,, \tag{S3}$$

$$S_c = 417 \times 8 = 3{,}336\,, \tag{S4}$$

$$V_{pr} = 21 \times 96 \times 10\,\mu\text{L} = 20{,}160\,\mu\text{L}\,, \tag{S5}$$

$$S_{pr} = 21 \times 96 = 2{,}016\,, \tag{S6}$$

$$V_{pc} = 20 \times 96 \times 10\,\mu\text{L} = 19{,}200\,\mu\text{L}\,, \tag{S7}$$

$$S_{pc} = 20 \times 96 = 1{,}920\,. \tag{S8}$$

When generating the pool amplicon libraries, we extract genomic DNA from 2 mL of culture from each pool, irrespective of its total volume. This means the volume of each species in a row pool that is processed through genomic DNA extraction is,

$$v_r = 2\,\text{mL}/S_r = 396.8\,\text{nL}\,. \tag{S9}$$

Similarly, the volumes of each species that go through genomic DNA extraction from any column, plate row or plate column pool are,

$$v_c = 2\,\text{mL}/S_c = 599.5\,\text{nL}\,, \tag{S10}$$

$$v_{pr} = 2\,\text{mL}/S_{pr} = 992.0\,\text{nL}\,, \tag{S11}$$

$$v_{pc} = 2\,\text{mL}/S_{pc} = 1{,}041.7\,\text{nL}\,. \tag{S12}$$

As we do not normalize the quantity of template loaded into any of the nested PCR reactions used for amplicon library generation (**Methods**) the amount of template of any individual species will be proportional to the volume of that species that was processed by genomic DNA extraction.

While the exact number of amplicons produced by a mutant will depend upon the sequence of the gene disrupted in that mutant, it is likely that the average number of amplicons (and hence later reads) generated per species is a function of the amount of template per species in the amplicon generation reaction. While it is important to remember that the amplicon generation reaction is an exponential amplification process, so that the differences in template quantities will result in larger differences in product quantities, on average the ratio of products from two templates will be at least as great as the ratio

of their templates. Thus, we expect that on average the ratio of amplicons (and hence reads) produced by any species in a column pool and a row pool will be,

$$\left\langle r_r / r_c \right\rangle \leq v_r / v_c = S_c / S_r = 8/12 = 0.667 \quad . \tag{S13}$$

Similarly,

$$\left\langle r_{pr} / r_{pc} \right\rangle \leq S_{pc} / S_{pr} = 1{,}920/2{,}016 = 20/21 = 0.952 \quad , \tag{S14}$$

$$\left\langle r_c / r_{pr} \right\rangle \leq S_{pr} / S_c = 2{,}016/3{,}336 = 0.604 \quad , \tag{S15}$$

$$\left\langle r_c / r_{pc} \right\rangle \leq S_{pc} / S_c = 1{,}920/3{,}336 = 0.576 \quad , \tag{S16}$$

$$\left\langle r_r / r_{pr} \right\rangle \leq S_r / S_{pr} = 2{,}016/5{,}040 = 0.4 \quad , \tag{S17}$$

$$\left\langle r_r / r_{pc} \right\rangle \leq S_{pc} / S_r = 1{,}920/5{,}040 = 0.381 \quad . \tag{S18}$$

We have marked these maximum expected ratios on the distributions in **Fig. 3C-H** of the main text.

Additionally, the expected centers of the distributions of read counts for each of the pool axes, relative to the center of the row pool read counts are,

$$\left\langle r_c \right\rangle \geq 12 \left\langle r_r \right\rangle / 8 = 1.5 \left\langle r_r \right\rangle \quad , \tag{S19}$$

$$\left\langle r_{pr} \right\rangle \geq 139 \left\langle r_r \right\rangle / 56 = 2.48 \left\langle r_r \right\rangle \quad , \tag{S20}$$

$$\left\langle r_{pc} \right\rangle = 21 \left\langle r_r \right\rangle / 8 = 2.63 \left\langle r_r \right\rangle \quad . \tag{S21}$$

The expected minimum centers of the read count distributions are marked in **Supplementary Fig. 4**.

Supplementary Note 3: Simulation of Read Count Ratios

We used Monte Carlo simulations to understand the distributions of the ratios of read counts between the four pool axes for sequences that mapped to single addresses in the *S. oneidensis* progenitor collection.

We noted that the read counts for the single-address-mapping lines in the pool presence table are approximately log-normally distributed (**Supplementary Fig. 4**) and that the centers of these distributions increase as the amount of template per species used in the amplicon generation reactions is increased (**Supplementary Note 2**). For instance, the center of the read count distribution for column pool coordinates is should be at least 1.5× that of the center of the row pool coordinates (**Supplementary Fig. 4B; Equation S19**).

We constructed a Monte Carlo model of two correlated log-normally distributed sets of numbers, *r* and *c*. Initially, both sets of numbers have the same mean and standard deviation (**Supplementary Fig. 5A**). The distribution of ratios also follows a log-normal function with a center at 1 (ln(1) = 0) (**Supplementary Fig. 5B**).

The ratios of the means of the distributions are then adjusted by multiplying the column count by 12/8.

$$\bar{c} = 12\bar{r}/8 \quad \text{(S22)}$$

This has the effect of moving the center of the distribution of ratios to a lower value (ln(8/12) = -0.4) (**Supplementary Fig. 5D**).

The distributions are then further correlated by the application of a covariance matrix;

$$\begin{bmatrix} \bar{r}_2 \\ \bar{c}_2 \end{bmatrix} = \begin{bmatrix} 1 & 0 \\ 0.7 & 0.714 \end{bmatrix} \begin{bmatrix} \bar{r} \\ \bar{c} \end{bmatrix} \quad \text{(S23)}$$

This has the effect of shifting the ratio distribution to even lower values (**Supplementary Fig. 5F**), much as is observed in the real read count ratios (**Fig. 3C-H**).

Finally, we applied a threshold to the random distributions that eliminated pairs of read counts unless both read counts were higher than 5. This has no detectable effect on the fits (**Supplementary Fig. 5H**).

A code (KOSUDOKU-RANDOMRATIOS) to implement this simulation is included in the KOSUDOKU package.

Supplementary References

1. Giaever, G. & Nislow, C. The yeast deletion collection: a decade of functional genomics. *Genetics* **197,** 451–465 (2014).

2. Baba, T. *et al*. Construction of *Escherichia coli* K-12 in-frame, single-gene knockout mutants: the Keio collection. *Mol Syst Biol* **2,** 2006.0008 (2006).

3. Manoil, C. Tagging exported proteins using *Escherichia coli* alkaline phosphatase gene fusions. *Meth Enzymol* **326,** 35–47 (2000).

4. Goodman, A. L. *et al*. Identifying Genetic Determinants Needed to Establish a Human Gut Symbiont in Its Habitat. *Cell Host & Microbe* **6,** 279–289 (2009).

5. Goodman, A. L. *et al*. Extensive personal human gut microbiota culture collections characterized and manipulated in gnotobiotic mice. *Proceedings of the National Academy of Sciences* **108,** 6252–6257 (2011).
